# Development of renewable energy fed three-level hybrid active filter for EV charging station load using Jaya grey wolf optimization

**DOI:** 10.1038/s41598-024-54550-7

**Published:** 2024-02-23

**Authors:** Koganti Srilakshmi, D. Teja Santosh, Alapati Ramadevi, Praveen Kumar Balachandran, Ganesh Prasad Reddy, Aravindhababu Palanivelu, Ilhami Colak, C. Dhanamjayulu, Ravi Kumar Chinthaginjala, Baseem Khan

**Affiliations:** 1 Department of Electrical and Electronics Engineering, Sreenidhi Institute of Science and Technology, Hyderabad, TS 501301 India; 2 CVR College of Engineering/Computer Science and Engineering, Hyderabad, India; 3 Department of Electrical and Electronic Engineering, Velagapudi Ramakrishna Siddhartha Engineering College, Kanuru, Vijayawada, Andhra Pradesh India; 4 Department of Electrical and Electronics Engineering, Vardhaman College of Engineering, Hyderabad, TS 501218 India; 5 Department of Electrical and Electronics Engineering, AM Reddy Memeorial College of Engineering, Guntur, AP India; 6 Department of Electrical Engineering, Annamalai University, Chidambaram, Tamil Nadu India; 7 Department of Electrical and Electronics Engineering, Faculty of Engineering and Architectures, Nisantasi University, 34398 Istanbul, Turkey; 8 School of Electrical Engineering, Vellore Institute of Technology, Vellore, India; 9 School of Electronics Engineering, Vellore Institute of Technology, Vellore, Tamil Nadu India; 10 Department of Electrical and Computer Engineering, Hawassa University, P.O. Box 05, Hawassa, Ethiopia

**Keywords:** Hybrid power filter, Fractional order proportional integral controller, Harmonic distortion, Energy science and technology, Engineering

## Abstract

This work develops a hybrid active power filter (HAPF) in this article to operate in conjunction with the energy storage system (ESS), wind power generation system (WPGS), and solar energy system (SES). It employs three level shunt voltage source converters (VSC) connected to the DC-bus. Optimization of the gain values of the fractional-order proportional integral derivative controller (FOPIDC) and parameter values of the HAPF is achieved using the Jaya grey wolf hybrid algorithm (GWJA). The primary objectives of this study, aimed at enhancing power quality (PQ), include: (1) ensuring swift stabilization of DC link capacitor voltage (DCLCV); (2) reducing harmonics and improving power factor (PF); (3) maintaining satisfactory performance under different combinations of loads like EV charging load, non linear load and solar irradiation conditions. The proposed controller's performance is evaluated through three test scenarios featuring different load configurations and irradiation levels. Additionally, the HAPF is subjected to design using other optimization algorithms such as genetic algorithm (GA), particle swarm optimization (PSO), and ant colony optimization (ACO) to assess their respective contributions to PQ improvement.

## Introduction

In order to lessen the strain on converters and ratings, the integration of renewable energy sources such as solar and wind power into the distribution network has been promoted in recent years. A conventional square-wave inverter produces a square wave with an extensive amount of harmonics as its output. Filters must be used in order to customize the output and give it a sinusoidal shape. The cost and size of the filter increase when using typical square-wave inverters, which is a major drawback. The excellent characteristics of multi-level inverters result in output that is level. Leveled output needs less filtering than conventional square-wave inverters.

### Motivation

The importance of active power filters in distributed power generation systems and micro-grids has recently gained attention. The incorporation of shunt active power filter (SHAPF) with sustainable energy sources has grown progressively more significant in contrast to the conventional grid-connected VSC. Among the many advantages of this approach are the ability to maintain a stable DCLCV in the face of load fluctuations, increase PQ on the grid, protect sensitive loads from grid side disturbances, and improve the converter's fault ride-through capability during transient events. Notably, 3-phase, 3-wire distribution systems are especially well-suited for the use of shunt filters in conjunction with renewable energy sources. The creation of reference signals is one important component of SHAPF. Most of the methods in the literature now in publications are traditional PI controller, sliding mode controller, and artificial intelligence techniques with varying loads; nevertheless, they failed to acquire the optimized values using metaheuristic algorithms, performance analysis of system during irradiation, and load variations with multilevel VSCs for multi-objective optimization.

### Literature survey

A new, automated transition mechanism between grid and island modes was developed for the solar-battery integrated UPQC to mitigate power quality issues. Furthermore, the system's performance was verified through experimental results^1^. The solar-integrated UPQC using the artificial neuro fuzzy interface system (ANFIS) technique was developed to enhance power quality, and an evaluation of its performance was conducted under various load and supply conditions^2^. Besides, UPQC integrated with PV systems using an adaptive compensation technique was suggested for employing the variable leaky least mean square algorithm, to eliminates a low-pass or moving average filter to extract fundamental components from the distorted source voltage and load current and to generates the reference signal for controlling the switching of both shunt and series voltage source converter (VSC) in the UPQC^3^. Meanwhile, a multi-objective computational challenge was introduced to focus on determining the best locations and configurations for grid-connected PV systems to optimize the power generation reliability for various scenarios involving different generation probabilities^4^.

The adaptive distributed power control technique was suggested to handle the issues like THD and voltage distortions by adopting two H-connected configurations, each equipped with eight switches, for the 3-φ UPQC^5^. Further, the PPDM was introduced for the UPQC for multilevel Cascaded Inverter. Its primary goal is to mitigate voltage fluctuations (sag and swell), address current harmonics, and ensure the consistent maintenance of the DCLCV^6^. Moreover, a multi-level cascade UPQC was designed to reduce supply voltage distortions and THD by harnessing PV, wind, and fuel cell as power sources^8^.

A UPQC system incorporating PV and battery energy storage was recommended to reduce THD and address grid voltage issues^9^. Besides, the several control strategies and algorithms for UPQC were explored regarding PQ enhancement, ultimately leading to the recommendation of a versatile control approach^10^. However, the Solar Battery-based Synchronous Uninterruptible Active Power Filter was introduced to regulate reactive power and reduce THD of current waveforms efficiently. Additionally to produce suitable reference currents, the Maxikalman filter was developed^11^. However, for voltage regulation and reactive power control within the grid, an artificial neural network (ANN) controller that relies on feed-forward training was applied to a UPQC system connected to wind and solar sources^12^.

The UPQC was utilized to rectify voltage imbalances, mitigate current harmonic distortions, and enhance network efficiency. This was achieved by implementing an ANFIS control^13^. The FLC was recommended for the series filter integrated into the grid-tie three phase distribution system to address PQ issues such as voltage irregularities, reduction of current signal THD, and the consistent maintenance of DCLCV^14^. However, an SRFT scheme based PIC was specifically crafted to the fuel cell associated synchronous uninterruptible active power filter. The primary objective aims to reduce the current harmonics and regulation of DCLCV^15^. The proposal included implementing intelligent Fuzzy-PI and Fuzzy-PID controllers within an AC-DC microgrid to tackle PQ issues and improve voltage stability. Furthermore, the controllers' efficiency was showcased through two scenarios with varying loads^16^.

The recommendation was made to enhance UPQC using an adaptive full-order technique aimed to accurately identifying faults under various dynamic load changes and grid conditions. As well, a BBO metaheuristic method was applied to select gain parameters of PIC for stabilizing DC-Link oscillations effectively^17^. Besides the experimental configuration of an fullbridge DC-DC converter was studied in conjunction with snubber circuit^7^. A hybrid control method was devised, which combines the Improved Bat Algorithm and Moth Flame Algorithm for addressing power quality issues within a micro-grid system. This approach aims to minimize the error function associated with power variations. Optimally tuning the K_p_ and K_i_ parameters helps reduce the operational costs of renewable energy sources^18^. A hybrid control system utilizing fuzzy back-propagation was implemented for a 5-level UPQC to reduce THD and enhance power factor^19^. Furthermore, a novel approach integrating sequence-component detection with unit vector-template generation was suggested for the double-stage solar-integrated UPQC, aimed at mitigating power quality issues^20^. The novel social based grey wolf hunting behavior based metaheuristic algorithm was developed to solve multi objective engineering problems^21^. On other hand, the jaya algorithm was developed for address engineering issues effectively^22^.

A Hybrid Shunt Active Power Filter was optimized using a combination of Particle Swarm and Grey Wolf Optimization techniques, along with FOPIDC, to effectively compensate for reactive power and harmonic distortion in both balanced and unbalanced loading scenarios. Here, the parameters of the FOPID controller were adjusted using the PSO-GWO technique in order to reduce the impact of harmonics^23^. Besides, a novel study was introduced for generating switching pulses using adaptive fuzzy hysteresis current-regulated hybrid shunt active power filter. This approach aims to compensate for reactive power and harmonics in distribution networks^24^. A strategy for compensating harmonics was presented for hybrid AC-DC interlinking converters to reduce the switching frequencies. The discourse encompassed the proposed approach, modeling methodologies, stability assessment, and precise virtual impedance formulation^25^.

However, a cost-effective Hybrid Shunt Active Power Filter was designed to compensate for harmonics and reactive power. The Robust Extended Complex Kalman Filter technique is employed once more to estimate the per-unit in-phase fundamental component of the reference current. The maximum value of the reference current is achieved by implementing a novel Self-Adaptive Fuzzy-PID Controller. The settings of the suggested controller are optimized using an Improved Football Game Optimization technique^26^. The hybrid shunt active power filter (HSAPF) was presented that utilizes dual-tree complex wavelet transform (DT-CWT) for reference current calculation. Additionally, conventional proportional-integral (CPI), type-1 fuzzy logic controller (T1FLC), and type-2 fuzzy logic controller (T2FLC) were employed to optimize the parameters of the HSAPF. The objective was to improve the harmonic compensation capability and power factor^27^.

The shunt active power filter aims to generally lower the THD and might not eliminate selective harmonic components, whereas the passive power filter removes specific harmonic components. The HAPF reduces total THD while also eliminating specific harmonics. Numerous conventional HAPF design techniques lack global optimal design parameters and do not make advantage of optimization algorithms. The process of extracting reference signals from source and load currents and voltages, as well as the dynamic behavior of selected controllers, are critical to the efficient operation of HAPF which requires precise mathematical models. The HAPF's performance will also reduce due to parameter changes, load-side disturbances, nonlinear behavior, and other factors. Most often, a trial-and-error method is used to alter the HAPF controller parameters, which might not provide the greatest performances.

### Key contribution

The following steps emphasize the novelty of this manuscript:

- Presenting the novel HAPF with optimized filter parameters with the developed nature-inspired metaheuristic GWJA.
- Optimization of FOPIDC gains parameters for cascading H-bridge 3L-VSC with the developed hybrid GWJA in order to minimize defects in the current waveforms.
- Integration of SES, WPGS, and ESS to the 3L-Shunt filter VSC's DC connection to minimize the strain and stress on the filter, help it meet load demands, and keep the DCLCV constant during load and radiation fluctuations.
- The suggested approach aims to improve PF, maintain steady DCLCV in a short amount of time, and reduce source current THD. To further demonstrate the recommended method's performance, it is tested in three scenarios with different combination of loads like EV charging station, non liner, solar power generation, and wind speed variation.
- Lastly, the performance analysis of developed GWJA with GA, PSO, and ACO algorithms was carried out to show its viability.

This work is organized as follows: "Design and modeling of proposed system" section provides the 3L-Shunt filter VSC design and modelling; "Control scheme" section outlines the suggested control scheme; "Simulation and results" section provides the results along with discussions; and "Conclusion" section concludes the manuscript.

## Design and modeling of proposed system

**Figure 1:**
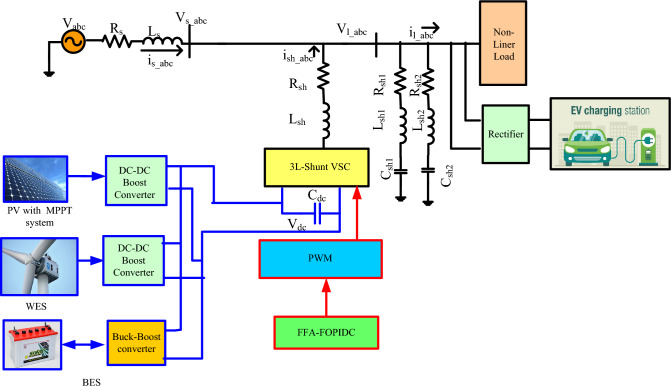
3L-shunt VSC based HAPF with charging station load.

The suggested 3L-Shunt filter VSC architecture, which links the ESS, WPGS, and SPS to the SHAPF's DC connection, is depicted in Fig. 1. The interface inductance is what connects the HAPF to the grid. The HAPF aims to reduce the current waveform harmonics and maintain DCLCV constant with a short settling time by injecting an adequate compensatory current. One of the most effective configurations is the cascaded H-Bridge topology for multi-level inverters, which eliminates the need for clamping tools or components. While the layout of the cascaded H-Bridge topology requires multiple DC sources to supply each individual H-Bridge cell with power.

**Figure 2:**
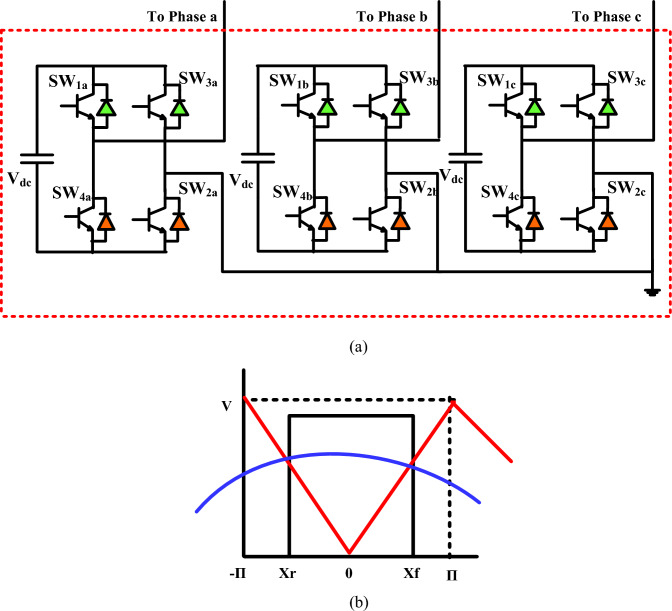
3L-cascade H bridge VSC. (**a**) 3L-cascade H bridge shunt VSC, (**b**) Three level with PWM

This design incorporates the arrangement of two H-Bridge cells in a cascaded configuration, resulting in the formation of a three-level H-Bridge structure. Each H-Bridge cell is independently powered by its dedicated DC source. Figure 2a displays the configuration of a three-level cascaded H-Bridge multi-level inverter. The sequential activation of power switches facilitates the achievement of a three-level output as the total DC link voltage is distributed across the H-Bridge cells. Furthermore, the arrangement and switching order of the power switches used in the three-level cascaded H-Bridge are visually represented in Table 1.

**Table 1:** Switches' ON/OFF states for the 3L-cascaded Hbridge VSC.

Output voltage	SW1	SW2	SW3	SW4
$V_{dc/2}$	On	On	Off	Off
0	Off	On	Off	On
$- V_{dc/2}$	Off	Off	On	On

### Modelling of three level VSC

**Figure 3:**
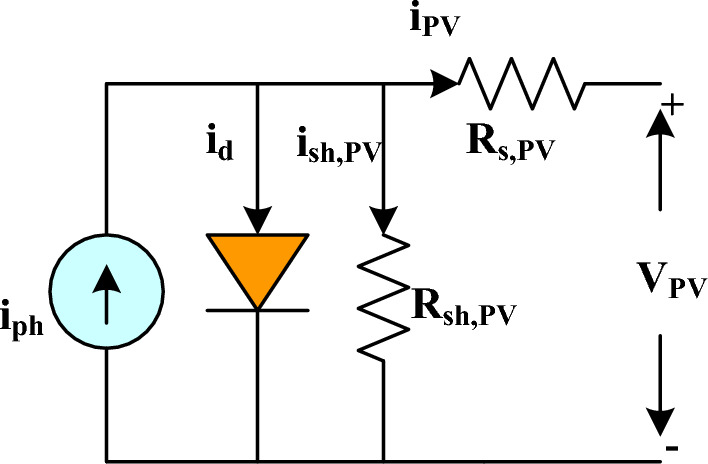
PV single cell model.

The 3-level converter necessitates two level-shifted carriers that are in synchronization and each of them has a peak magnitude of V_dc_ with a frequency of ω_c_, where ω_0_ represents the frequency of the reference signal. Figure 3 displays the waveform of the output produced by the 3-level inverter. Switching occurs based on the naturally sampled carriers in relation to the reference signal. The idea of Fourier decomposition states that every periodic waveform v(t) that varies over time can be represented as an infinite series of harmonics. That is to say,

1 $$ v(t) = a_{0} /2 + \sum\limits_{m = 1}^{\infty } {a_{m} \cos mt + b_{m} \sin mt} $$

A function v(x, y) of two variables can be defined as:

2 $$ \begin{gathered} x(t) = \omega_{c} t + \theta_{c} \hfill \\ y(t) = \omega_{0} t + \theta_{0} \hfill \\ \end{gathered} $$

Using two variables x and y, Eq. (1) can be generalized as:

3 $$ v(x,y) = a_{0} (y)/2 + \sum\limits_{m = 1}^{\infty } {a_{m} (y)\cos mx + b_{m} (y)\sin mx} $$

Figure 2b depicts a single-pulse of a three-level inverter that is produced by naturally sampled PWM. In modulation, the reference waveform is compared with the carrier waveform. The period of the carrier signal is represented by 2π. X_r_ and X_f_ represent the ascending and descending edges, respectively, of the generated pulse. These edges are measured from the center of the carrier waveform. Their definitions are as follows:

4 $$ \begin{gathered} X_{f} = + \Pi \times M_{i} \cos y \hfill \\ X_{r} = - \Pi \times M_{i} \cos y \hfill \\ Where,M_{i} = \frac{{V_{m} }}{V} \hfill \\ \end{gathered} $$

5 $$ \begin{aligned} a_{0} & = 2 \times M_{i} \times \cos y \\ am & = \frac{2}{\pi m}\sin (Mi \times m \times \pi \times \cos y) \\ b_{p} & = 0 \\ \end{aligned} $$

By substituting the value of (4–5) into Eq. (3), a generalized expression can be obtained. Let m represent the baseband index, p represent the sideband index.

### Design of shunt active power filter

The main purpose of a shunt filter is to provide a supply current that is free from distortion by injecting the required amount of current at the point of common coupling (PCC). The control circuit utilizes Eq. (6) to calculate the required magnitude of injected current.

6 $$ i_{s} = i_{l} - i_{sh} $$

7 $$ V_{s} = V_{m} \sin wt $$

8 $$ \begin{aligned} i_{l} & = \sum\limits_{n = 1}^{\infty } {i_{n} \sin } (nwt + \phi_{n} ) \\ & = i_{1} \sin (wt + \phi_{1} ) + \sum\limits_{n = 1}^{\infty } {i_{n} \sin } (nwt + \phi_{n} ) \\ \end{aligned} $$

9 $$ P_{l} = V_{s} *i_{l} $$

Equation (10) can be used to get the value of *C*_*dc*_

10 $$ C_{dc} = \frac{{\pi *i_{sh} \;}}{{\sqrt 3 \omega V_{cr,pp} }} $$

The $V^{ref}_{dc}$ is chosen as 700 V. Peak to peak voltage ripple ($V_{cr,pp}$) and shunt compensatory current determine will decide best C_dc_. The shunt inductance value (*L*_*sh*_) connects the shunt VSC to the network, and it depends on the dc-link voltage (V_dc_), switching frequency, and ripple current as follows:

11 $$ L_{sh} = \frac{{\sqrt 3 \;m\;V_{dc} }}{{12\;a_{f} \;f_{sh} \;I_{cr,pp} }} $$

The value of depends on peak to peak ripples ($I_{cr,pp}$), assuming that, overloading factor ($a_{f}$) is 1.5, modulation depth ($m$) is 1, and the switching frequency ($f_{sh}$) is 10 kHz.

### Modelling of external components

The diode clamped 3L-VSC is suggested to use the SPS, WPGS, and ESS fed DC link. The DCLCV is controlled by a renewable source with ESS support during changes in load. By reducing the demands made by the utility, external supply sources help to decrease the converter ratings and stress. Equation (12) provides the DC link power demand ($P_{dc}$) for the proposed approach.

12 $$ P_{PV} + P_{W} + P_{BSS} = P_{dc} $$

#### Solar PV system (SPS)

The Simulink library is used for the PV model that was used in this investigation. The PV modules are linked together in series to form a string to create the necessary quantity of voltage and current. Following that, some of these strings are joined in parallel. Figure 3 gives the single-diode circuit model.

The constituents of it consist of resistances (*R*_*s,PV*_ and* R*_*sh,PV*_) through which the current (*i*_*PV*_*, i*_*sh,PV*_) flows, as well as a photocurrent (*i*_*ph*_) accompanied by a forward diode current (*i*_*d*_). The PV cell detects solar energy and converts it into electrical current. The PV current (*i*_*PV*_) can be calculated using Kirchhoff's Current Law (KCL), as demonstrated by Eq. (13).

13 $$ i_{PV} = i_{ph} - i_{d} - i_{sh} $$

The PV modules are connected in parallel and series to form an array using Eq. (14).

14 $$ i_{{_{PV,m} }} = i_{ph} N_{p} - i_{S,PV} N_{p} \left[ {\exp \left( {\frac{{Q(V_{PV} + N_{s} /N_{p} (i_{PV,m} R_{S,PV} ))}}{{N_{s} \eta kT_{C} }}} \right) - 1} \right] - \frac{{V_{PV,m} + N_{s} /N_{p} (i_{PV,m} R_{S,PV} )}}{{N_{s} /N_{p} (R_{sh,PV} )}} $$

where

15 $$ i_{ph} = (i_{ph,n} + K_{1} \Delta T_{C} )\frac{G}{{G_{n} }} $$

**Figure 4:**
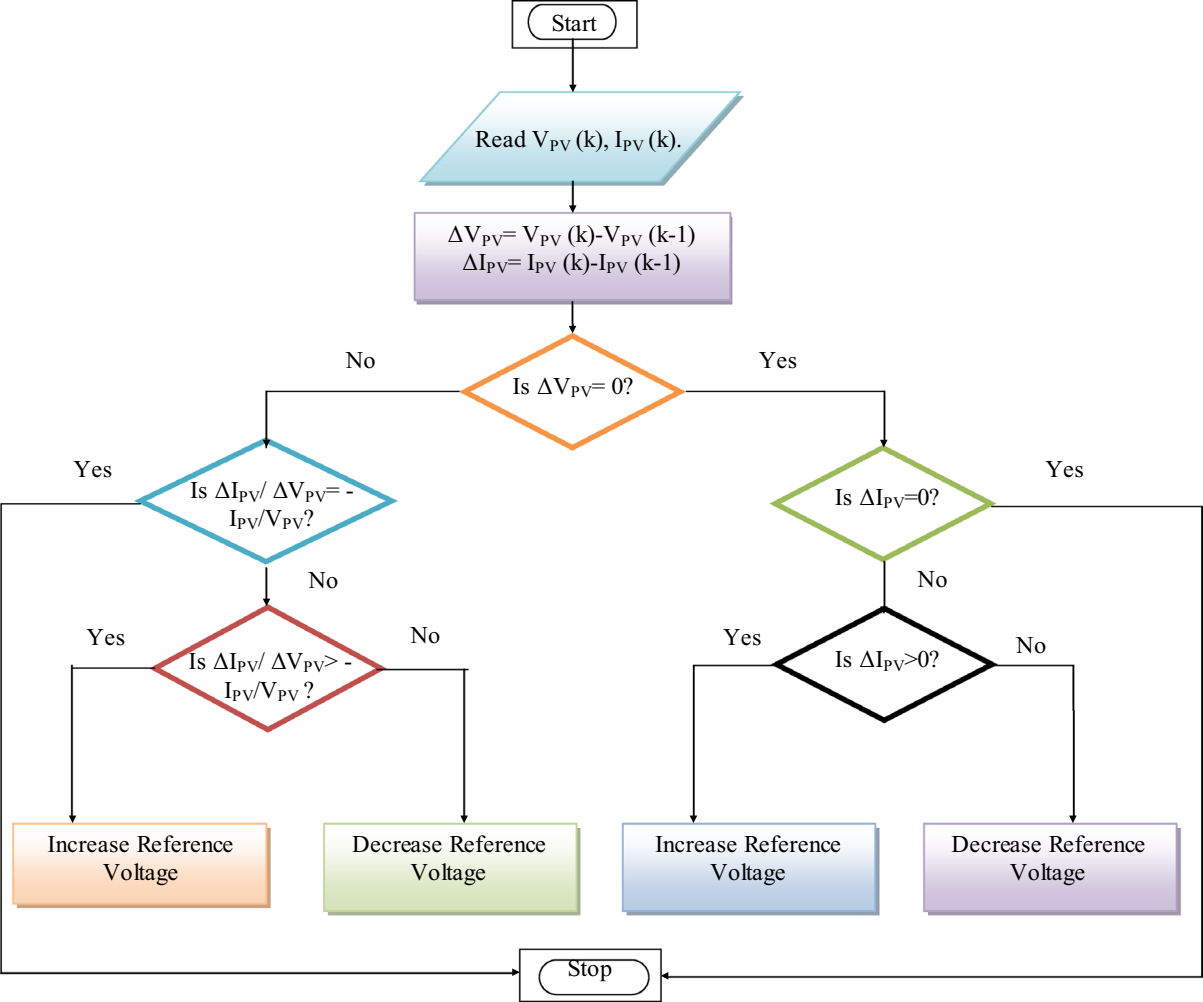
Incremental conductance MPPT.

This study utilized the incremental conductance (INC) technique to enhance the photovoltaic efficiency by employing the MPPT approach. The INC method works by continuously monitoring the output power of the solar panel and adjusting the operating point to maximize power output. The algorithm utilizes the incremental conductance (∆I/∆V) of the solar panel, where ∆I is the change in current and ∆V is the change in voltage. The algorithm compares the INC to a reference value to determine the direction in which the operating point should be adjusted shown in Fig. 4. The INC approach provides several benefits, including rapid reaction to changing conditions, precise tracking even under partial shadowing, high efficiency, simplicity, and compatibility with different PV technologies. Consequently, it is widely favored as the MPPT technique in solar power systems. Equation (16) provides a description of the solar output.

16 $$ P_{PV} = V_{PV} \times i_{PV} $$

#### ESS

**Figure 5:**
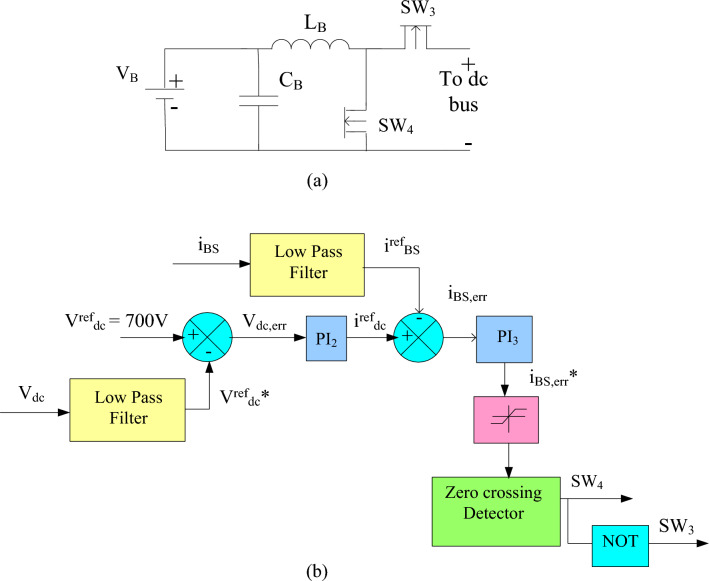
ESS control. (**a**) ESS configuration at DC link, (**b**) ESS controller

The ESS incorporates a Li-ion energy storage battery, which has many benefits like, low discharge, and minimal number repairs. The battery can be charged or discharged via switches SW3 and SW4, as shown in Fig. 5a. The battery's state-of-charge (SOCB) is represented by Eq. (17).

17 $$ SOCB = 100(1 + \int {i_{BS} \;} dtQ) $$

The battery's charging or discharging is determined by the amount of SPS and WPGS the SOCB limit defined in Eq. (18).

18 $$ SOCB_{\min } \le SOCB \le SOCB_{\max } $$

Here, *Q* and $i_{BS}$ represent the battery and current, whereas *SOC *_*max*_ and *SOC *_*min*_ indicate the allowable highest and lowest values for SOCB. The control circuit of battery operation w.r.to the required DC link voltage is shown in Fig. 5b.

#### WPG S

**Figure 6:**
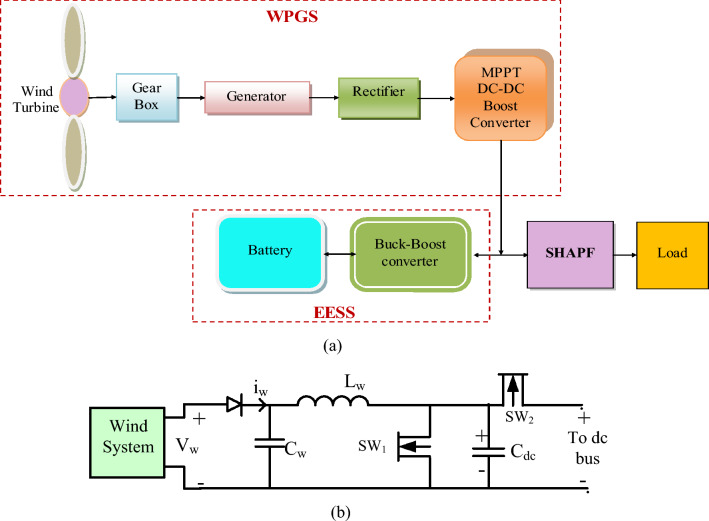
WPGS at DC bus support. (**a**) schematic, (**b**) WPGS configuration at DC link

The AC voltage produced by the wind is converted into DC voltage. This DC voltage is then amplified using buck-boost converter (BBC) consisting of switches and various components, as illustrated in Fig. 6a,b. The proposed concept utilizes a wind turbine equipped with a permanent magnet synchronous generator, chosen for its benefits of low maintenance requirements and reduced operational expenses. The equation for WPGS is represented by Eqs. (19) to (23).

19 $$ P_{m} = 1/2\pi \;\rho \;C_{p} (\lambda ,\beta )\;R^{2} \;V^{3} $$

20 $$ \lambda = \frac{{\omega_{m} R}}{v} $$

21 $$ \omega_{m} = \omega_{t} \;G_{r} $$

22 $$ C_{p} (\lambda ,\beta ) = 0.23\;\left( {\frac{116}{{\lambda_{1} }} - 0.48\beta - 5} \right)\exp^{{\frac{ - 12.5}{{\lambda_{1} }}}} $$

23 $$ \lambda_{1} = \left( {\frac{1}{{\frac{1}{\lambda - 0.02\beta } - \frac{0.0035}{{3\beta + 1}}}}} \right) $$

where $\rho$ denotes air density (1.225 kg/m^3^), $P_{m}$ indicated mechanical power, $\beta$ represents Pitch angle, $v$ signifies the wind velocity (m/s), R represents blade radius, $\omega_{m}$ indicates rotational speed of the rotor,$\lambda$ gives the tip-speed ratio, $G_{r}$,$\lambda_{1}$ is the gear ratio and constant, $\omega_{t}$ signifies wind plant angular speed, $C_{p}$ indicates power coefficient. Table 2 provides the power management at DC link of renewable sources and Table 3 presents the list of SPS, WPGS, and ESS values chosen for the work.

**Table 2:** Power dispersion at DC link.

Modes of operation	Action taken
Mode-1: When No *SPG* &* WPG*	BES only will provide power to $P_{DC}$
Mode-2: When *SPG* & *WPG* = $P_{DC}$	Solar PV and wind will supply power $P_{DC}$
Mode-3: When *SPG* & *WPG* < $P_{DC}$	The difference sum of the power will be provided by Battery till it reaches $SOCB_{\min }$
Mode-4: When *SPG* & *WPG* > $P_{DC}$	Excessive solar power is used to charge ESS system till it reaches $SOCB_{\max }$

**Table 3:** External sources specifications.

Equipment	Factor	Value chosen
PV panel	PV cells connected in parallel, series	66, 5
	Rated Power	305.226W
	Current under short circuit	5.98A
	Voltage under open circuit	64.2 V
	Current and voltage under max power	54.7 V /5.58A
Li-ion battery	Voltage at full charge condition	350 V
	Rated Capacity	350Ah
Wind turbine	mechanical power of turbine	4 MW
	Base power of the generator	400e3/0.9
	wind velocity	11 m/sec

## Control scheme

The key goals of SHAF are to stabilize the DCLCV to a constant value and reduce waveform defects by injecting an appropriate amount of current. It carries out the following frame transformations: (i) dq0 and abc; (ii) GWJA-based optimal selection of FOPDIC and HAPF values is chosen to meet required goals. Since the literature survey already provides access to the ABC and dq0 conversions, the control method of the proposed GWJA-based FOPID is emphasized here.

### FOPIDC

Equation (19) provides the transfer function for the suggested FOPIDC.

24 $$ TF = K_{p} + \frac{{K_{i} }}{{S^{\lambda } }} + K_{d} S^{\mu } $$

**Figure 7:**
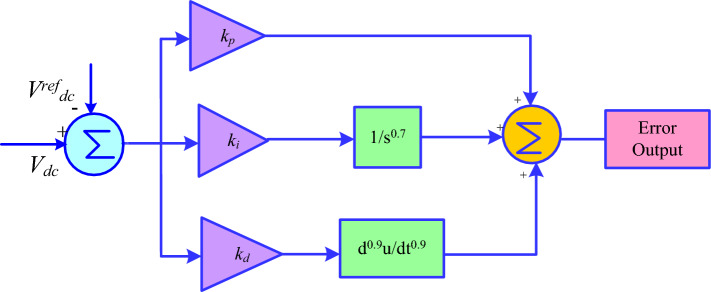
FOPIDC.

In this case, K_p_, K_i_, and K_d_ stand for the proportional, integral, and derivative gains of the suggested FOPIDC, respectively, and λ and µ indicate the fractional orders of the integrator and differentiator. Figure 7 displays the FOPIDC's control. However, FOPIDC helps in reducing the steady state error, thus makes the system more stable compared to other techniques.

The loadcurrent is transformed into the dq0 frame using the pharos technique, while the frequency is determined by the grid voltage via PLL. The ability of the SHAPF is dependent upon the generation of the reference current and the regulation of the DCLCV. Nevertheless, if the load fluctuates, it can cause changes in power flow, resulting in DCLCV variation. In order to stabilize the DCLCV, it is necessary for the active power in SHAPF should be equivalent with the switching losses. The GWJA-tuned FOPIDC injects a error, which is determined by calculating the difference between the reference and actual DC Link voltages using Eq. (25).

25 $$ \Delta i_{dc} = e_{1} (t) = V^{ref}_{dc} - V_{dc} (t) $$

**Figure 8:**
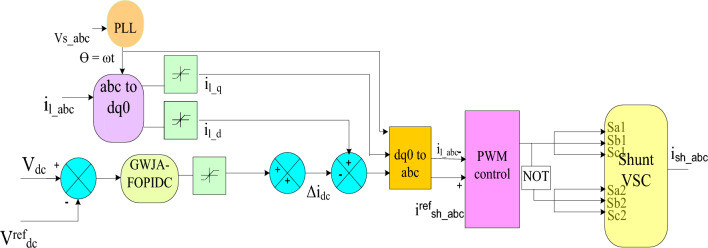
Hybrid Controller for Shunt Converter.

The dth part of the load currentis combined with the error derived from FOPIDC. The dq0 elements are translated to the abc framework and then compared with the load current using a PWM controller to provide the required gate signals. The control of SHAPF with the suggested controller is illustrated in Fig. 8.

### Proposed hybrid optimization algorithm

#### Conventional grey wolf optimization

The hunting behaviour and headship hierarchy of grey wolves are described by the GWO^21^ algorithm. The three primary wolves dedicated to the hunting task are denoted as *α*, *β*, and *δ*. Among these wolves, *α* is regarded as the leader who makes judgements about the hunting process, where to sleep, when to wake up, etc. *β* and *δ*, on the other hand, represent the second and third levels and assist α in making decisions. Furthermore, the last stage of the wolf is devoted as $\zeta$ with eating. The surrounding features are modelled using Eqs. (26) and (27), where *M* and *L* stand for coefficient vectors, *J*_P_ for prey position vectors, *J* for grey wolf position vectors, and it for the current iteration. The model for *M* and *L* is shown by Eqs. (28) and (29), where $a$ is a parameter that is continuously minimised from 2 to 0 throughout the course of the iterations. The random vectors that fall between [0, 1] are indicated by *ra*_1_ and *ra*_2_, while the maximum iteration is indicated by *it*_max_.

26 $$ D = \left| {L.J_{p} \left( {it} \right) - J\left( {it} \right)} \right| $$

27 $$ J\left( {it + 1} \right) = J_{p} \left( {it} \right) - M.D $$

28 $$ M = 2\overset{\lower0.5em\hbox{$\smash{\scriptscriptstyle\frown}$}}{a} .ra_{1} - \overset{\lower0.5em\hbox{$\smash{\scriptscriptstyle\frown}$}}{a} $$

29 $$ L = 2ra_{2} $$

The numerical mechanism from Eq. (30) to Eq. (35), which specifies the final location updating assessment, is the formula for characterising the hunting behaviour of wolves by Eq. (36). Algorithm 1 specifies the standard GWO model's pseudo code.

30 $$ D_{\alpha } = \left| {L_{1} .J_{\alpha } - J} \right| $$

31 $$ D_{\beta } = \left| {L_{2} .J_{\beta } - J} \right| $$

32 $$ D_{\delta } = \left| {L_{3} .J_{\delta } - J} \right| $$

33 $$ J_{1} = J_{\alpha } - M_{1} .\left( {D_{\alpha } } \right) $$

34 $$ J_{2} = J_{\beta } - M_{2} .\left( {D_{\beta } } \right) $$

35 $$ J_{3} = J_{\delta } - M_{3} .\left( {D_{\delta } } \right) $$

36 $$ J\left( {it + 1} \right) = \frac{{J_{1} + J_{2} + J_{3} }}{3} $$

**Figure:**
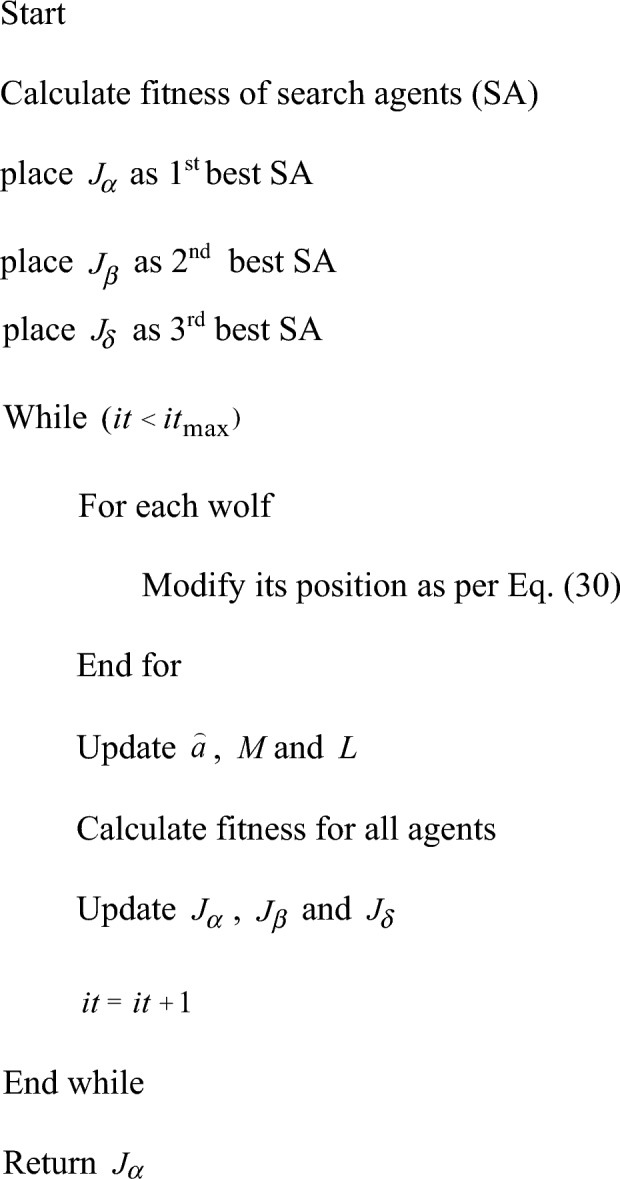
Algorithm 1 Standard GWO

#### Jaya optimization algorithm

JA^22^ is predicated on the idea that the best solution should be prioritised over the worst solution while addressing a given problem. Consider that the population size is "$P$" and the number of modelling constraints is " ‘$V$’(i.e. $p = 1,2,...V$) " at each *i*th iteration. If $J_{p,r,i}$ represents the *r*th aspirant's *p*th factor value over the course of the *i*th iteration. Additionally, this value is modified in accordance with Eq. (37), which $J_{p,best,i}$ indicates the value of the limitation for the optimal result and $J_{p,worst,i}$ as the value of the *p*th constraint when taking the population's worst answer into account.

37 $$ J^{\prime }_{p,r,i} = J_{p,r,i} + r_{1} \left( {J_{p,best,i} - \left| {J_{p,r,i} } \right|} \right) - r_{2} \left( {J_{p,worst,i} - \left| {J_{p,r,i} } \right|} \right) $$

Additionally, $J^{\prime }_{p,r,i}$ indicates the updated value of, $J_{p,r,i}$, $r_{1}$, and $r_{2}$, which are arbitrary values within the interval [0,1]. The phrase "$r_{1} \left( {J_{p,best,i} - \left| {J_{p,r,i} } \right|} \right)$" indicates that the solution is trying to go in the direction of the best solution, and the component “− $r_{2} \left( {J_{p,worst,i} - \left| {J_{p,r,i} } \right|} \right)$” shows that it is trying to move away from the worst option. $J^{\prime }_{p,r,i}$ is accepted if the function value it produces is enhanced.

#### Developed GWJA model

In this study, a new hybrid method that chooses the best optimal value of PIC is developed. Actually, there are problems with the traditional GWO model that impact the optimisation process, such as poor searching performance, poor precision, and slower convergence. Similarly, the JA algorithm should be enhanced in terms of its convergence speed. Hence, the integration of both approaches is intended to enhance convergence and yield superior results. Here, the GWO model and the JA concept are combined. The main reason for selecting hybrid algorithm is that the disadvantages of one algorithm are overcome by other algorithm.

**Figure 9:**
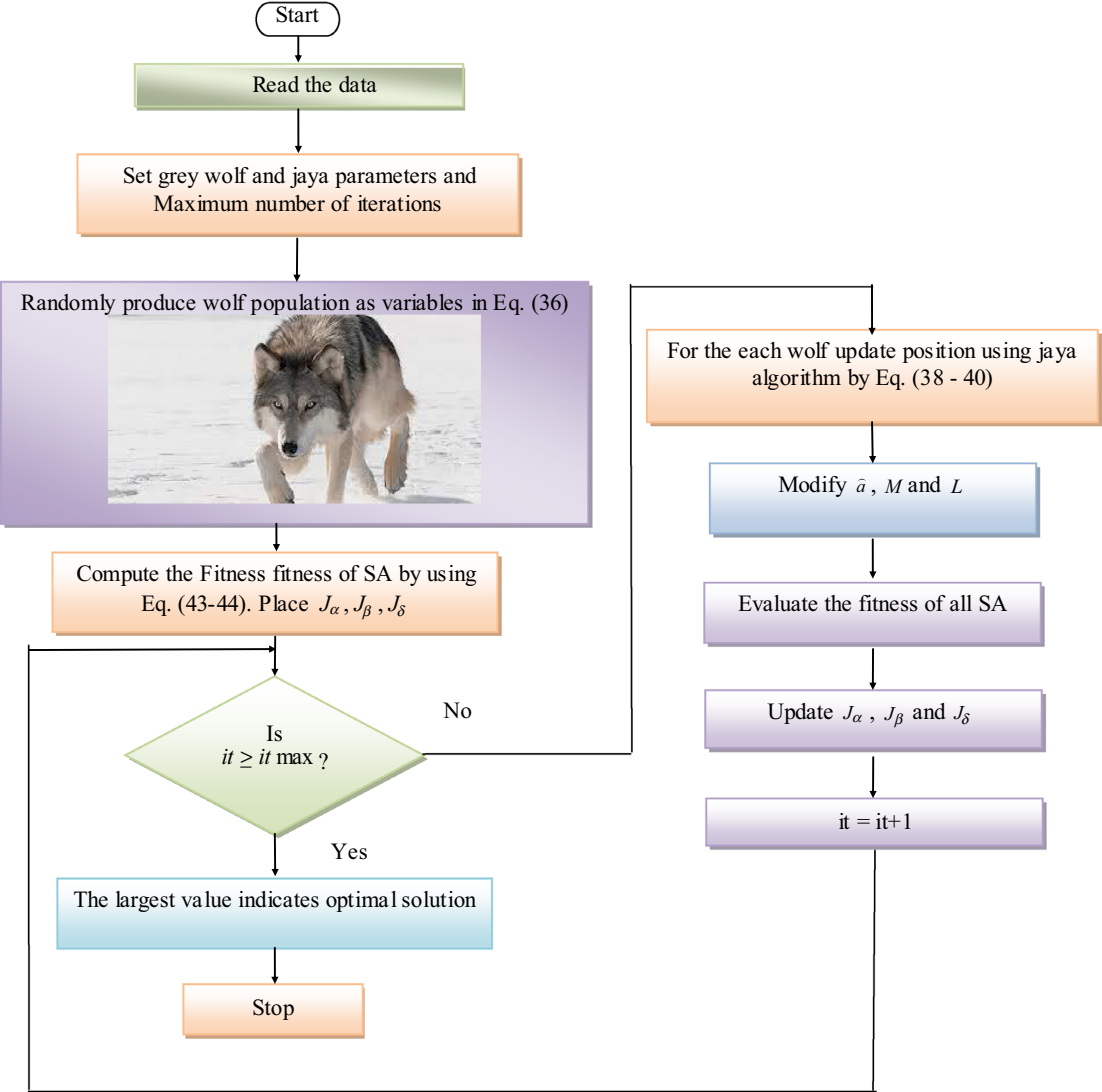
Developed GWJA flow Chart.

According to Eqs. (33)–(35), the position of and is updated in the traditional GWO. On the other hand, the suggested algorithm updates the GWO positions using the formulas found in Eqs. (38)–(40). The suggested algorithm is called the GWJA model since it integrates the JA concept with the GWO paradigm. Algorithm 2 provides the pseudocode for the described GWJA model, whereas Fig. 9 provides a flowchart depiction of the model. In this work the paramters of GWJA is selected on trial and error method which are selected as such SA as 8, [0,1] gives search domain, runs for each optimizer are considered as 20, α as 0.99, β as 0.01 with max 150 iterations.

38 $$ J_{1} = J + r_{1} \left( {J_{\alpha } - \left| J \right|} \right) - r_{2} \left( {J_{p,worst,i} - \left| J \right|} \right) $$

39 $$ J_{2} = J + r_{1} \left( {J_{\beta } - \left| J \right|} \right) - r_{2} \left( {J_{p,worst,i} - \left| J \right|} \right) $$

40 $$ J_{3} = J + r_{1} \left( {J_{\delta } - \left| J \right|} \right) - r_{2} \left( {J_{p,worst,i} - \left| J \right|} \right) $$

**Figure:**
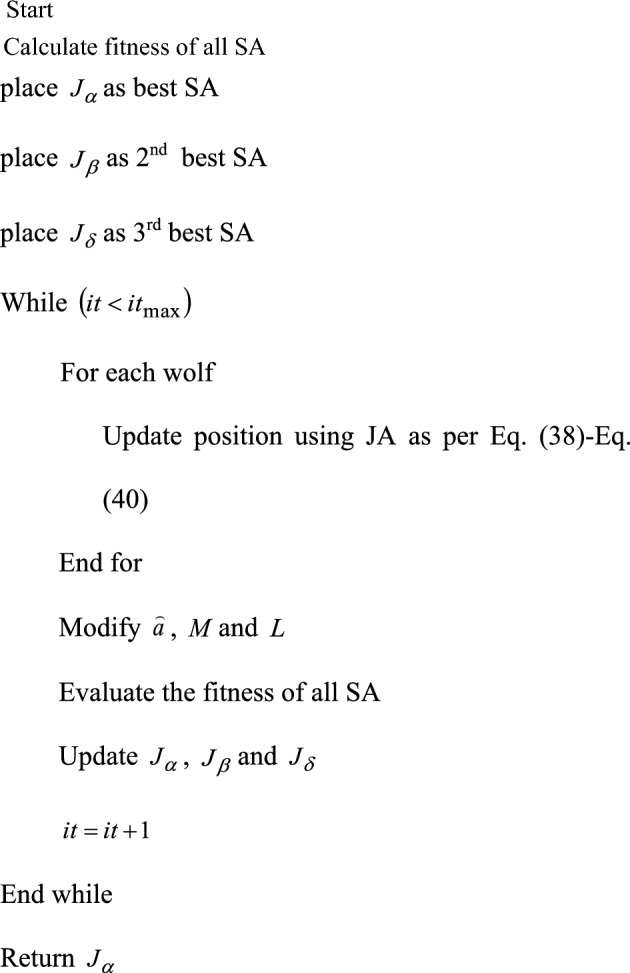
Algorithm 2 Proposed GWJA

#### Representation of problem variables

Initially, it is essential to compute the fitness function, which serves as the goal function. The grey wolf population should be set up which represents the solution to the problem by Eq. (41).

41 $$ J_{i} = [ \, K_{{\text{p}}} {\text{, K}}_{{\text{i}}} {\text{, K}}_{{\text{d}}} {, }\lambda {,}\mu ,R_{sh} ,L_{sh} ,R_{sh1} ,L_{sh1} ,R_{sh2} ,L_{sh2} ,C_{sh1} ,C_{sh2} ] $$

The limitations are shown as

42 $$ J_{i} (\min ) \le J_{i} \le J_{i} (\max );i = 1,2...n $$

where n serves as number of design variables. The bonds of the chosen control parameters were listed in Table 5.

43 $$ Maxf = \frac{1}{1 + THD} $$

where

**Table 5:** Lower, Upper bonds of FOPIDC and filter variables.

Decision variable	_Kp_	_Ki_	_Kd_	_λ_	_µ_	_Rsh (Ω)_	_Rsh1 (Ω)_	_Rsh2(Ω)_	_Lsh (mH)_	_Lsh1 (mH)_	_Lsh2 (mH)_	_Csh1 (µF)_	_Csh2 (µF)_
Lower	0	0	0	0	0	0	0	0	0	0	0	0	0
Upper	100	100	100	2	2	1	1	1	1	10	10	100	100

THD is be evaluated by Eq. (44)

44 $$ THD = \frac{{\sqrt {(I^{2}_{2} + } I^{2}_{3} + .....I^{2}_{n} )}}{{I_{1} }} $$

## Simulation and results

A 3-phase distribution system with a variety of harmonic loads, such as changing wind speed and irradiation, was used to test the presented approach. The Simulink model for the suggested system and the test system were created in the Matlab 2016a framework. Table 6 provides a list of the system's different parameters. In order to examine the performance of the GWJA trained FOPIDC based SHAPF, three test scenarios with various harmonic load blends, including EV load, non linear load unbalanced load, fixed and variable irradiation, and wind velocity, were selected and provided in Table 7. For every test case, with and without SHAPF, the system's power factor (PF) and source current THDs were recorded and compared to the most widely used PIC, SMC, and FLC in Tables 8 and 9, respectively. The suggested system's waveforms for examples 1–3 are shown in Figs. 11, 12, and 13. These represent the supply/grid (V_s_), load (V_l_), dc-link (V_dc_) voltages, as well as the load (i_l_), injection (i_sh_), and supply (i_s_) currents, irradiation (G), temperature (T), and wind velocity (W).

**Table 6:** System with SHAPF parameters.

Grid Supply	$V_{s}$: 415Volts ; $f$: 50Hertz
DClink	$C_{dc}$: 9400 microfarad ; $V^{ref}_{dc}$ = 700Volts

**Table 7:** Cases studies.

	Case study 1	Case study 2	Case study 3
Fixed (1000W/m2) solar irradiation	✓		
Variable irradiation		✓	✓
Fixed velocity of wind	✓	✓	
Variable velocity of wind			✓
Load-1: Balanced bridged rectifier non-linear load: 60Ω &30mH	✓		✓
Load 2: Unbalanced RL branch Load R_1_ = 10 Ω, R_2_ = 20 Ω &R_3_ = 15 Ω L_1_ = 1.5mH, L_2_ = 3.5mH &L_3_ = 2.5 mH		✓	✓
Load 3: EV charging station Load	✓	✓	✓

**Table 8:** Comparison of %THD.

Ref[]/Controller	Case-1	Case-2	Case-3
Without SHAPF	27.61	9.89	16.34
GA	4.224	4.987	4.336
PSO	3.598	3.875	3.001
ACO	3.001	2.434	3.445
^5^ PIC	4.01	–	–
^9^ PIC	2.62	–	–
^12^ ANN	3.52	–	–
^22^ ANFIS	2.43	–	–
^19^ ANN-Fuzzy	2.24	–	–
GWJA	2.16	2.12	2.04

**Table 9:** PF comparison.

Case	No SHAPF	GA	PSO	ACO	GWJA
1	0.6774	0.8879	0.9297	0.9924	0.9998
2	0.8124	0.9123	0.9613	0.9657	1
3	0.7314	0.8978	0.9854	0.9925	0.9997

**Figure 10:**
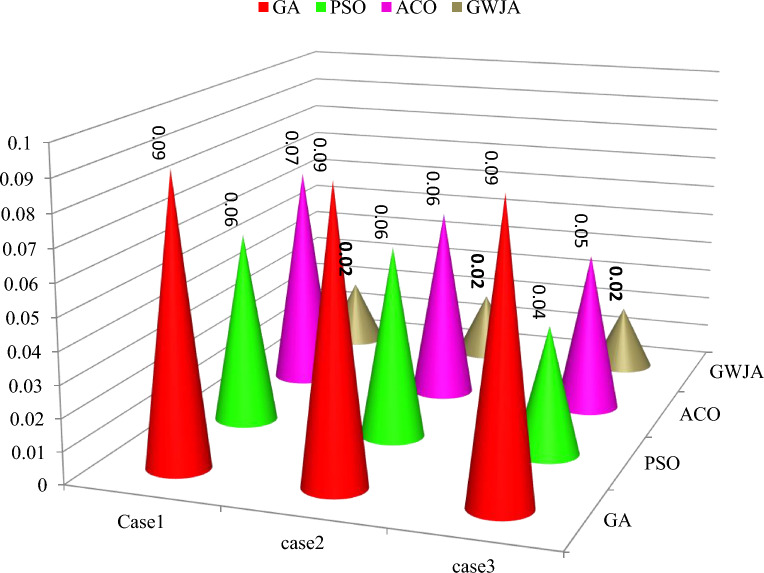
Comparison plot of time taken in sec to reach DCLCV stable.

**Figure 11:**
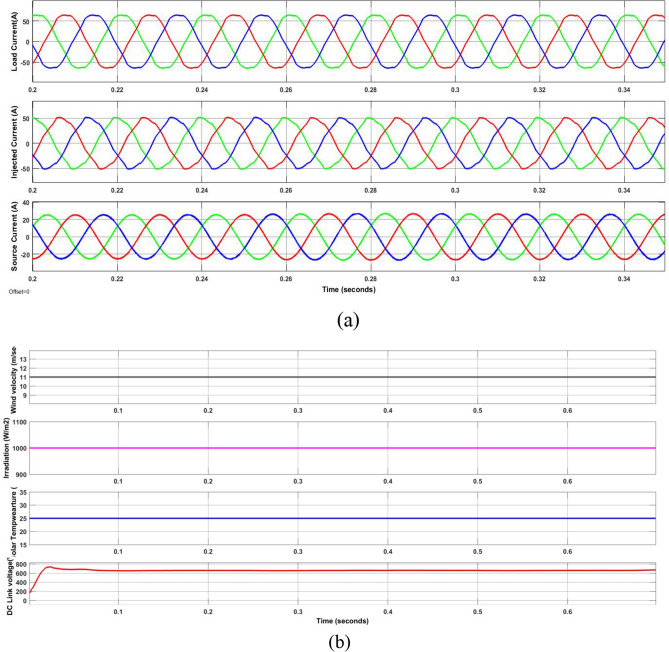
Waveforms of the suggested method for case one. (**a**) i_l_, i_sh_, i_s_, (**b**) G, T, velocity of wind, DLCV

As shown in Table 7 case 1 with the Load1 and Load2 resulted in a non-sinusoidal current at load which is balanced with a PF of 0.6774 and a THD of 27.61% (Fig. 10). The suggested approach may provide a harmonic free supply current, as Fig. 11a illustrates. Along with the current waveforms, there was also an improvement in the THD and PF values, which are displayed in Tables 8 and 9. By injecting the appropriate shunt currents, the PF boosted from 0.6774 to 0.998 and the THD of the load current decreased from 27.61 to 2.16%—lower than other approaches and literature procedures. As also shown in Fig. 11b, the suggested approach was able to rapidly get the DCLCV to a constant level in less than 0.02 s during fixed G, T and W. However, which is significantly less than other compared methods as listed in Table 8. Additionally, Table 10 provides the optimized values of the control parameters obtained with proposed GWJA and other standard optimization techniques like GA, PSO and ACO.

**Table 10:** Optimized values of control parameters of proposed GWJA, GA, PSO and ACO.

Case	_Method_	K_p_	K_i_	_Kd_	_λ_	_µ_	R_sh_(Ω)	R_sh1_ (Ω)	R_sh2_ (Ω)	L_sh1_ (mH)	L_sh_ (mH)	L_sh2_ (mH)	C_sh1_ (µF)	C_sh2_ (µF)
1	**GWJA**	**0.876**	**1.004**	**0.735**	**0.947**	**1.054**	**0.287**	**0.731**	**0.002**	**4.584**	**0.027**	**1.779**	**47**	**25**
	GA	1.498	3.001	0.997	0.887	1.132	0.314	0.401	0.012	5.889	0.117	2.114	53	28
	PSO	0.336	1.226	0.735	1.001	1.354	0.112	0.265	0.004	9.234	0.068	3.447	60	34
	ACO	1.912	2.374	0.874	0.981	0.987	0.003	0.197	0.017	6.968	0.901	4.001	71	38
2	**GWJA**	**2.998**	**42.631**	**1.664**	**0.5**	**1.00**	**0.110**	**0.942**	**0.036**	**4.185**	**0.018**	**2.319**	**58**	**29**
	GA	1.521	25.331	1.997	0.657	1.157	0.347	0.041	0.015	8.223	0.114	1.568	42	21
	PSO	2.112	29.740	0.223	1.203	0.887	0.051	0.354	0.354	7.965	0.097	5.124	51	23
	ACO	2.004	41.361	1.103	1.098	1.053	0.008	0.661	0.447	4.981	0.241	7.012	63	31
3	**GWJA**	**1.321**	**0.412**	**1.905**	**0.889**	**0.401**	**0.901**	**0.836**	**0.004**	**6.3317**	**0.748**	**2.082**	**47**	**28**
	GA	2.001	14.120	0.997	0.619	0.664	0.774	0.415	0.007	9.554	0.901	1.007	61	30
	PSO	1.998	13.981	1.998	0.165	1.003	0.442	0.034	0.010	7.991	0.005	2.365	78	39
	ACO	3.107	57.669	0.354	0.147	1.798	0.662	0.078	0.003	6.987	0.014	6.478	44	22

Significant values are in [bold].

**Figure 12:**
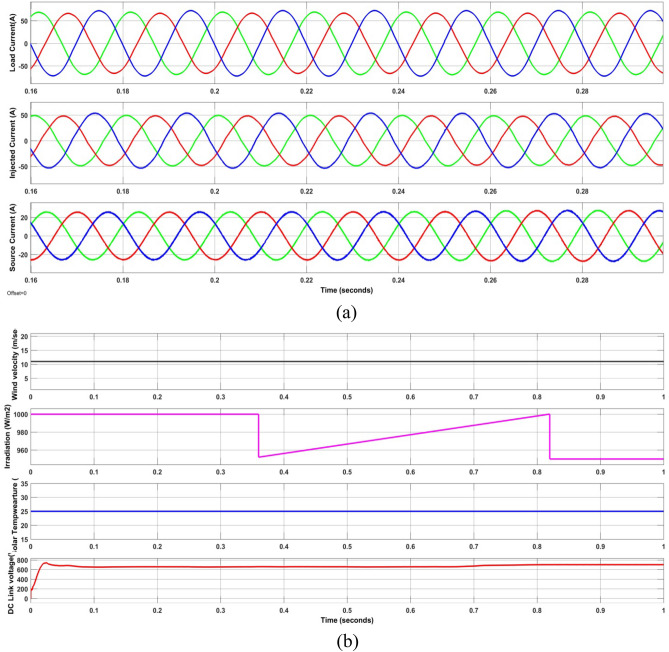
Waveforms of the suggested system for case two. (**a**) i_l_, i_sh_, i_s_, (**b**) G, T, velocity of wind, DLCV

As seen in Fig. 12a, the load current in case 2 was extremely imbalanced and sinusoidal. The power factor and load current had THDs of 0.8124 and 9.89%, respectively. Figure 12a illustrates how the proposed system was able to provide sinusoidal grid current by eliminating imperfections and introducing the appropriate quantity of shunt reactive current. The power factor rose to unity and the load current's THD dropped from 9.89 to 2.12%. However, the irradiation was constant till 0.35 s later if falls to 950W/m2 and further rises in ramp and reaches 1000 W/m^2^ at 0.85 s. It is evident that the GWJA-based FOPIDC quickly brings the DCLCV to constant 700 V, even during G, W fluctuation with T constant, is provided by Fig. 12b.

**Figure 13:**
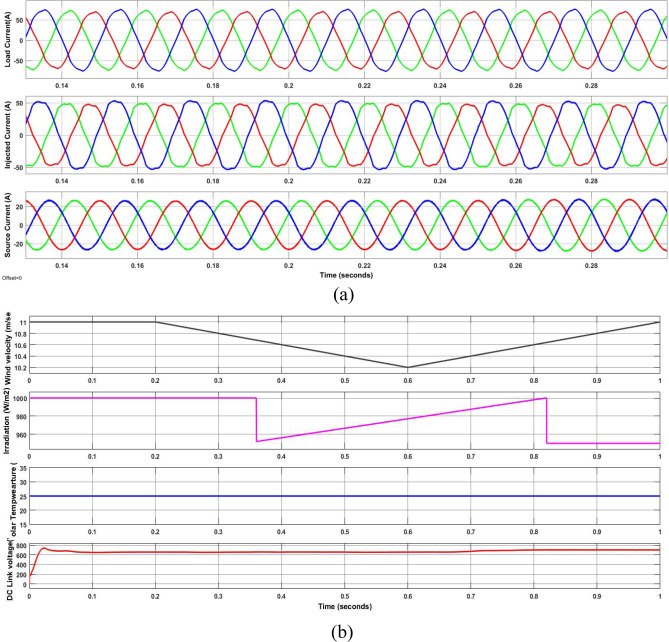
Waveforms of the suggested system for case three. (**a**) i_l_, i_sh_, i_s_, (**b**) G, T, velocity of wind, DLCV

**Figure 14:**
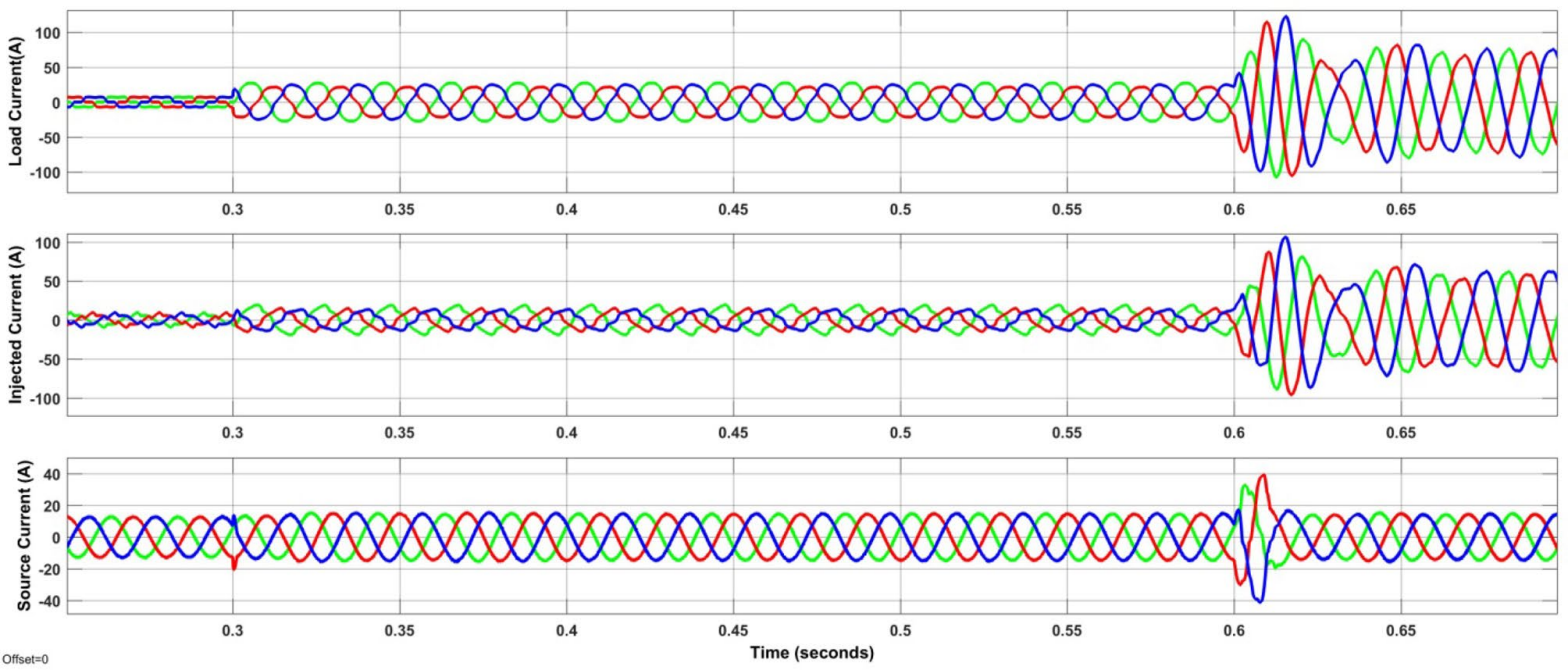
Waveforms of the dynamic load variation.

However, case-3 exhibits a comparable development in lowering the THD and raising the PF. The current at the load is observed to be non-sinusoidal with unbalance in phases due to Load 1, 2 and 3 acting simultaneously. From the Fig. 13a it is clear that the suggested method addresses the flaws in the current waveform effectively. Besides, the irradiation was constant till 0.35 s later if falls to 950 W/m^2^ and further rises in ramp and reaches 1000 W/m^2^ at 0.85 s. Similarly, the wind velocity is stable for 0.2 s and slowly falls to 10 m/sec at 0.6 s. Lastly, from Fig. 13b it is exhibited that even during load, irradiation and wind velocity variation the developed method maintains DCLCV stable. In this work, all the test cases undergo FFT analysis; however, Fig. 13 gives the outcomes of the dynamic behavior of test case 3, which comprised all kinds of non-linear and unbalanced loads with an EV charging station i.e. till 0.3 s rectifier bridge load alone however at 0.3 s unbalanced load is added to it and future at 0.6 s EV charging station load is additionally is connected (Fig. 14).

**Figure 15:**
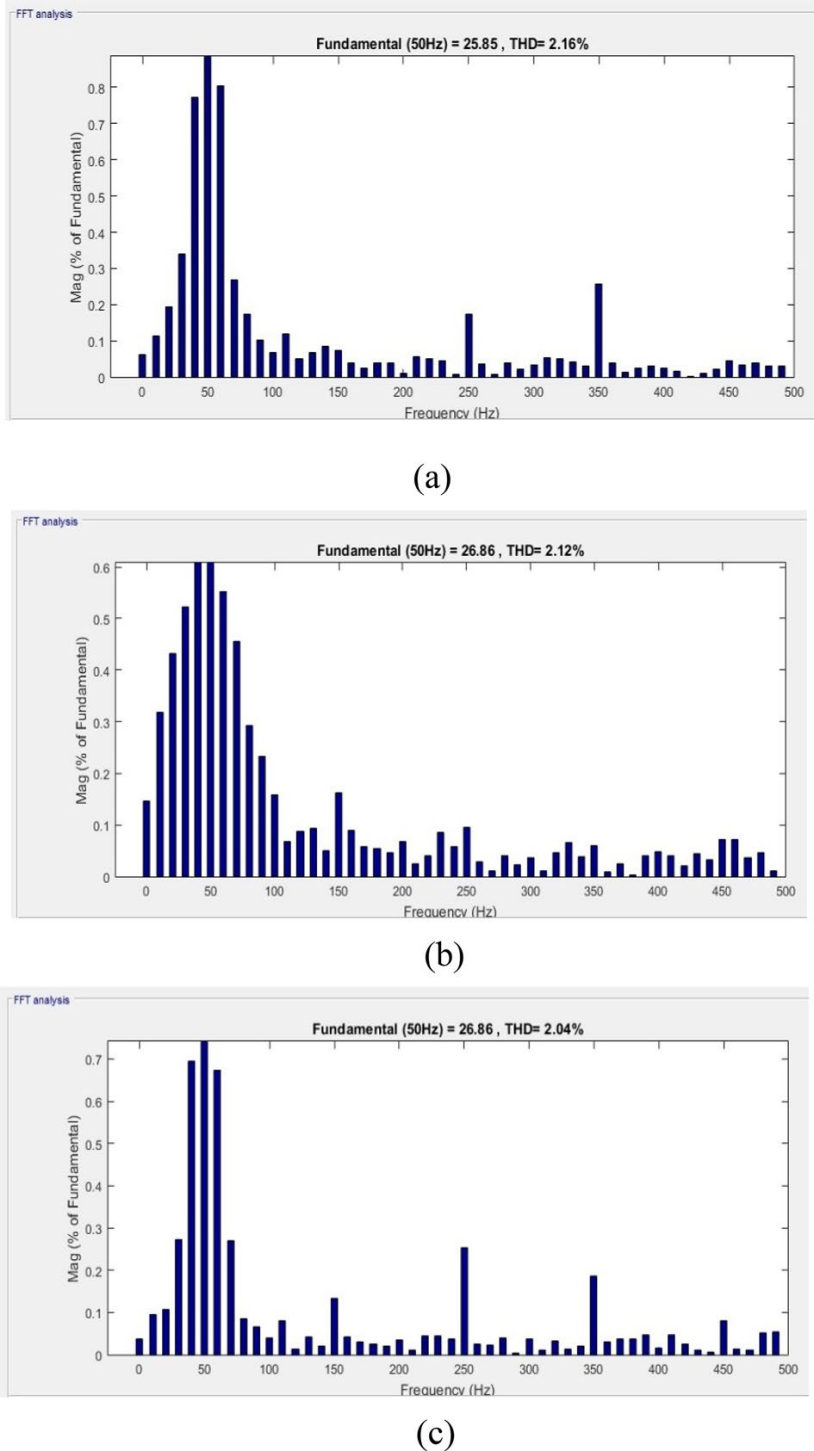
FFT spectrum for source current of all cases. (**a**) Case-1, (**b**) Case-2, (**c**) Case-3

**Figure 16:**
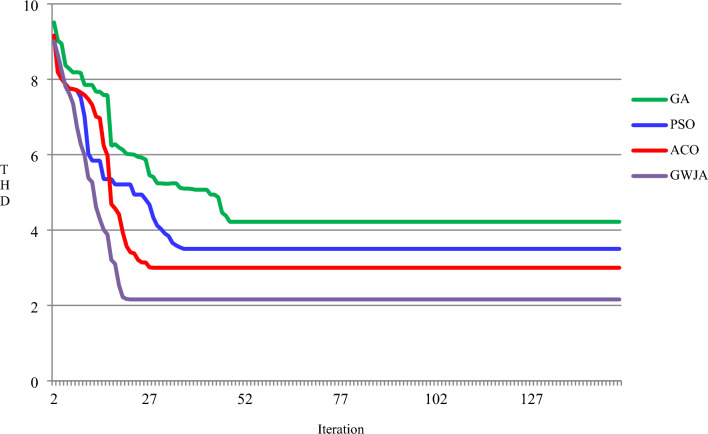
Convergence graph of case1.

The present harmonic spectra are displayed for each scenario in Fig. 15. Figure shows the measured time it took to reach the steady-state DCLCV and other controllers. It is evident from this that the created GWJA-tuned FOPIDC-based SHAPF only needs 0.02 s to reach steady-state dc-link voltage which is lesser than other methods as shown in Fig. 10. The discussion of results isan evident that the suggested technique works extremely well to lower THD, improve power factor, and maintain dc-link voltage stable with lower settling time. In addition from the Fig. 16 it is clearly noticeable that developed method converges in 22 iterations which is lesser that other algorithms GA at 48, PSO at 36, and ACO at 28. Efficiently managing the load and improving the power quality of electric vehicle charging stations is essential for maintaining grid stability, planning infrastructure, optimizing costs, and encouraging the wider use of electric vehicles. Integrating smart charging solutions with renewable energy sources and energy storage systems is crucial for accomplishing these aims.

## Conclusion

In this SHAPF project, a three-level shunt VSC was chosen. The GWJA was developed to optimize the selection of gain values for FOPIDC and HAPF parameters in three level shunt converters. Additionally, the modeling of several components such as SHAPF, solar, ESS, and WPGS was performed. This facilitated prompt intervention in adjusting the DCLCV, reducing the THD of source currents, and enhancing the power factor. Upon analyzing three test scenarios including diverse load configurations, such as rectifier bridge load, imbalanced load, and EV charging station load, with fixed and variable G and W, it is evident that the proposed controller effectively reduces THD to acceptable levels and significantly improves PF to nearly unity. The HAPF has been built using GA, PSO, and ACO to assess its effectiveness in terms of enhancing PQ. In addition, the suggested system demonstrated a faster attainment of stable DCLCV compared to alternative techniques. Subsequent research on the suggested model could focus mostly on artificial intelligence and deep learning techniques, as well as the latest metaheuristic optimization methods. Additionally, reduced switch multilevel VSC can also be considered as future work.

## Data Availability

The data used to support the findings of this study are included in the article.
